# Ray tracing optimization: a new method for intraocular lens power calculation in regular and irregular corneas

**DOI:** 10.1038/s41598-023-31525-8

**Published:** 2023-03-20

**Authors:** Pablo Pérez-Merino, Jaime Aramberri, Andrés Vásquez Quintero, Jos J. Rozema

**Affiliations:** 1 Centre for Microsystems Technology, Ghent University and Imec, Technologiepark 126, 9052 Ghent, Belgium; 2 Miranza Begitek, San Sebastian, Spain; 3 Miranza Okular, Vitoria, Spain; 4 Visual Optics Lab Antwerp (VOLANTIS), Faculty of Medicine and Health Sciences, University of Antwerp, Building T4, Universiteitsplein 1, 2610 Wilrijk, Belgium; 5 Department of Ophthalmology, Antwerp University Hospital, Wilrijkstraat 10, 2650 Edegem, Belgium

**Keywords:** Lens diseases, Refractive errors

## Abstract

To develop a novel algorithm based on ray tracing, simulated visual performance and through-focus optimization for an accurate intraocular lens (IOL) power calculation. Custom-developed algorithms for ray tracing optimization (RTO) were used to combine the natural corneal higher-order aberrations (HOAs) with multiple sphero-cylindrical corrections in 210 higher order statistical eye models for developing keratoconus. The magnitude of defocus and astigmatism producing the maximum Visual Strehl was considered as the optimal sphero-cylindrical target for IOL power calculation. Corneal astigmatism and the RMS HOAs ranged from − 0.64 ± 0.35D and 0.10 ± 0.04 μm (0-months) to − 3.15 ± 1.38D and 0.82 ± 0.47 μm (120-months). Defocus and astigmatism target was close to neutral for eyes with low amount of HOAs (0 and 12-months), where 91.66% of eyes agreed within ± 0.50D in IOL power calculation (RTO vs. SRK/T). However, corneas with higher amounts of HOAs presented greater visual improvement with an optimized target. In these eyes (24- to 120-months), only 18.05% of eyes agreed within ± 0.50D (RTO vs. SRK/T). The power difference exceeded 3D in 42.2% while the cylinder required adjustments larger than 3D in 18.4% of the cases. Certain amounts of lower and HOAs may interact favourably to improve visual performance, shifting therefore the refractive target for IOL power calculation.

## Introduction

Cataract is an age-related and vision-impairing disease characterized by a progressive loss of lenticular transparency, leading to a major deterioration in the retinal image quality. This condition is typically treated by replacing the crystalline lens with a foldable intraocular lens (IOL), eliminating the source of scattering while simultaneously providing a good correction for any remaining refractive errors. Traditional IOLs are monofocal with simple conic surfaces that are normally calculated for far vision, achieving almost far-emmetropic distance refraction in eyes with typical corneal shape and ocular dimensions^1,2^.

The postoperative optical quality of the eye is not only determined by the residual defocus, however, but also by the final amount of astigmatism and higher order aberrations (HOAs). For example, more than 0.75 diopters (D) of residual astigmatism in combination with HOAs might be visually significant, increasing blur and image degradation^3–5^. Therefore, to improve the visual quality and achieve the desired postoperative refractive outcome, it is critical to (i) individually select the appropriate IOL design and (ii) accurately calculate the IOL power and cylinder. Introducing more complex surface parameters into the IOL design allows more options to minimize corneal astigmatism and HOAs, prompting manufacturing companies to offer IOLs with complex aspherical and toric surfaces such as aspheric balance curves or transitional conic toric surfaces^6–8^. Regardless, there is still a great need to develop customized procedures for accurate IOL power calculations, especially for astigmatic correction using toric IOLs in eyes with different amounts of HOAs.

Toric IOLs are universally recommended in cataractous eyes with a corneal astigmatism higher than 1.5D, which is found in about 1 in 5 patients^9^. In clinical practice, many toric IOL power calculations are based on statistical regressions obtained from retrospective cases or theoretical formulas derived from paraxial optics. These approaches use axial distances and the toricity of the corneal curvature to determine the likely IOL position and its optimal power^10–14^. The refractive astigmatism after a toric IOL implantation often differs from the planned target of zero astigmatism, however, ranging between 0.3D and 1.8D in patients with regular corneal astigmatism^15,16^, and between 0.8D and 6.9D for the irregular corneas of patients with keratoconus^17,18^. This indicates that existing calculators may not always be able to accurately determine the most appropriate IOL power and cylinder values. Furthermore, complex IOL designs, such as multifocal or extended depth of focus, are ineffective in the presence of postoperative residual astigmatism or HOAs^5^.

Recent studies suggested another approach that selects the best possible IOL using a patient specific eye model and ray tracing. This methodology overcomes the limitations of current IOL power calculations by exploiting all information available in a corneal tomography measurement, such as the anterior and posterior corneal surface elevation instead of simplified parameters such as keratometric readings^19–24^. Consequently, these patient-specific models are gaining traction in the ophthalmological community. The ray tracing approach also allows predicting the postoperative refractive aberrations and retinal image quality, making it an ideal tool to calculate the interactions between the IOL power and cylinder and the natural corneal aberrations. This would help clinicians to first select the IOL type (either aspheric or toric) based on the corneal pattern, followed by choosing the right spherical and toric power while keeping the interactions between the defocus, astigmatism and HOAs in mind^25–27^.

The current study therefore proposes a ray tracing optimization (RTO) method to determine the optimal IOL power in regular and irregular corneal patterns using virtual ray tracing. To this end, the three-dimensional anterior and posterior corneal elevation data and axial distances were incorporated in custom computer eye models that allowed estimating the corneal and ocular aberrations, from which the retinal image quality could be derived. The following parameters were evaluated: (i) corneal astigmatism, (ii) corneal HOAs (e.g., spherical aberration, coma, trefoil, and secondary astigmatism) and (iii) through-focus Visual Strehl as optical quality metric. These parameters may be useful to evaluate the sphero-cylindrical correction that produced the highest visual performance in combination with different levels of HOAs and adjust current IOL calculations and surgical strategies. To our knowledge, this is the first work to report a methodology that optimizes the IOL selection to the natural higher order corneal aberrations.

## Material and methods

### Computational modelling

**Figure 1:**
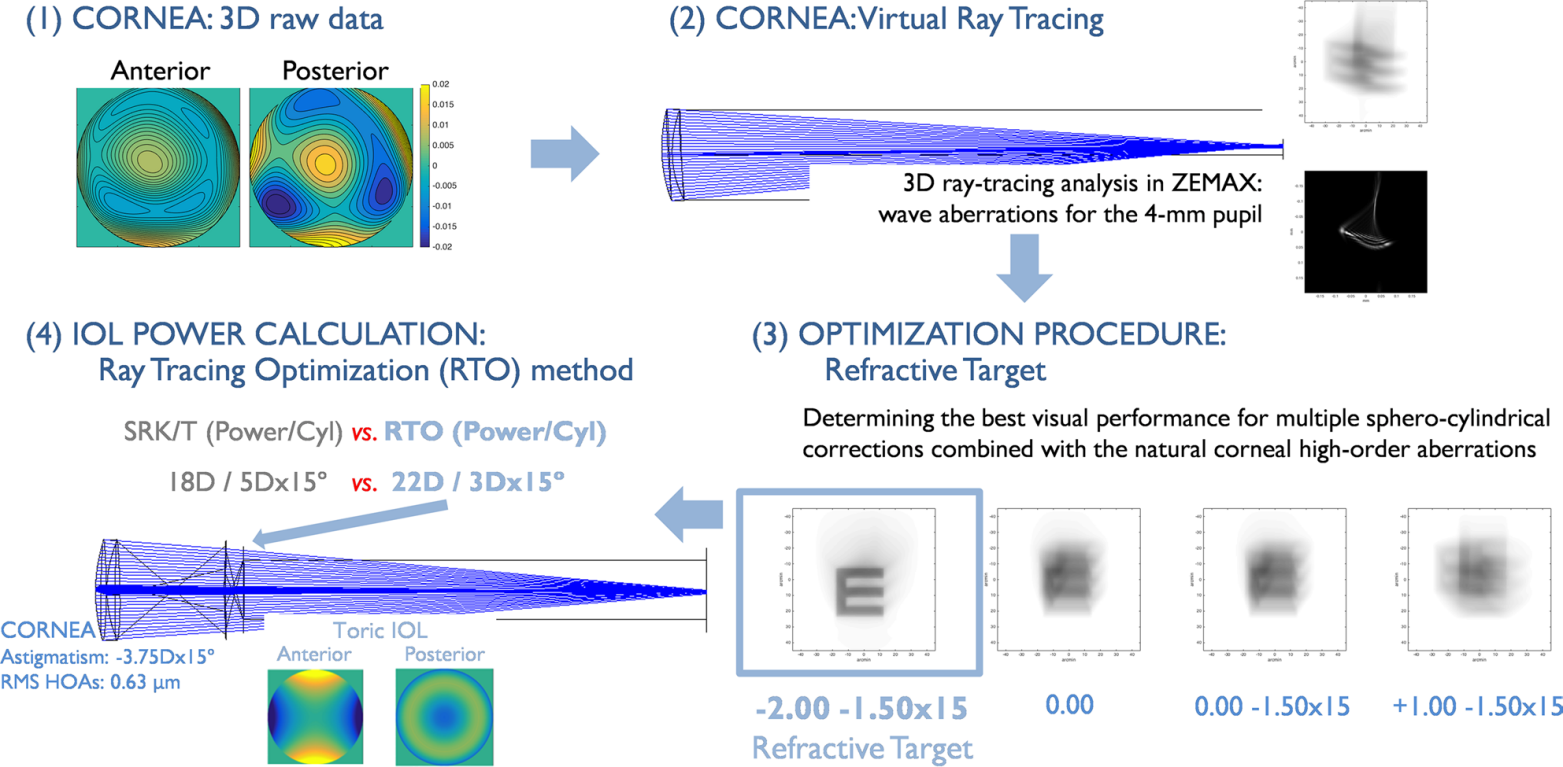
Illustration of the ray tracing optimization (RTO) method for IOL power calculation. (1) Corneal input data: anterior and posterior three-dimensional corneal elevation maps (SyntEye at 36 months; #145); (2) virtual ray tracing through the anterior and posterior corneal surfaces to determine the corneal wavefront aberrations at the pupil plane and the simulated visual performance (the point spread function, PSF, and the Snellen E-letter of 30 arc-min for 4-mm pupil diameter); (3) through-focus optimization process to calculate the sphero-cylindrical correction producing the highest VSOTF (representation of the Snellen E letter for different astigmatism and defocus magnitudes); and (4) pseudophakic virtual eye.

The steps of the computational modeling are illustrated in Fig. 1.

#### Step 1: From corneal surfaces to wave aberration

The elevation data of both anterior and posterior corneal surfaces of 210 higher order statistical eye models for developing keratoconus (SyntEyes KTC)^28^ were exported as Zernike polynomial expansions to ZEMAX (Focus Software, Tucson, Arizona, USA) for ray tracing analysis using Zernike Sag Surface type as input^29^. Note that Zernike sag surface in ZEMAX is in Noll´s format, so conversion is required from the OSA standards. The pupil diameter was 4 mm and fitted with a 28-term (6th order) Zernike expansion. Refractive indices of 1.376 and 1.334 were used for the cornea and aqueous humor, respectively. Wave aberrations for the central 4-mm pupil diameter area were calculated for monochromatic light of 555 nm by tracing an array of collimated rays through a 2-surface model of the cornea. The point source at infinity will be best focused on the retinal surface after iteration (best focus position). Wave aberrations were computed at the entrance pupil and described in terms of either individual Zernike coefficients (Zernike Standard Coefficients in ZEMAX) or the root-mean square (RMS) of astigmatism, coma, and trefoil.

SyntEyes at 0 and 12 months after simulated onset represent normal corneal patterns, while SyntEyes at 24, 36, 48, 60 and 120 months after simulated onset characterize mid- to late-stage keratoconus, with progressively irregular corneal surfaces and corneal thinning. The synthetic developing keratoconus model was validated with clinical data in a previous publication^28^. The average corneal parameters of different SyntEyes groups are listed in Table 1.

**Table 1:** Descriptive corneal parameters: corneal power (K1 and K2 in D), corneal astigmatism (D), the root-mean square (RMS) of corneal high-order aberrations (HOAs) and the spherical aberration of the cornea (in microns, μm; 4-mm pupil diameter). Average ± standard deviation [minimum, maximum]. # represents the eye number from the previous published SyntEyes KTC models^28^.

	K1 (D)	K2 (D)	Corneal astigmatism (D)	RMS HOAs (μm)	Spherical aberration (μm)
SyntEyes 0 months (n = 30, #001-030)	44.35 ± 1.49 [41.44, 48.56]	43.72 ± 1.57 [41.08, 48.12]	− 0.64 ± 0.35 [− 0.05, − 1.24]	0.10 ± 0.04 [0.04, 0.26]	0.04 ± 0.01 [− 0.01, 0.07]
SyntEyes 12 months (n = 30, #101-130)	44.46 ± 1.31 [42.30, 48.43]	43.86 ± 1.43 [41.17, 48.14]	− 0.61 ± 0.30 [− 0.09, − 1.45]	0.16 ± 0.08 [0.03, 0.38]	0.03 ± 0.02 [− 0.01, 0.07]
SyntEyes 24 months (n = 30, #031-060)	45.42 ± 2.48 [41.66, 54.86]	43.86 ± 2.44 [40.60, 53.68]	− 1.56 ± 0.93 [− 0.22, − 3.62]	0.48 ± 0.27 [0.16, 1.11]	− 0.01 ± 0.09 [− 0.33, 0.17]
SyntEyes 36 months (n = 30, #131-160)	45.98 ± 3.25 [40.67, 55.61]	43.62 ± 3.50 [37.52, 52.31]	− 2.36 ± 1.40 [− 0.22, − 5.96]	0.71 ± 0.36 [0.10, 1.51]	− 0.05 ± 0.18 [− 0.68, 0.19]
SyntEyes 48 months (n = 30, #061-090)	46.92 ± 3.24 [41.85, 52.90]	44.04 ± 3.05 [37.18, 49.77]	− 2.88 ± 1.28 [− 0.83, − 6.42]	0.80 ± 0.39 [0.22, 1.75]	− 0.05 ± 0.15 [− 0.49, 0.22]
SyntEyes 60 months (n = 30, #161-190)	46.51 ± 3.14 [41.95, 54.25]	43.17 ± 3.40 [37.16, 51.36]	− 3.33 ± 1.48 [− 0.68, − 6.12]	0.86 ± 0.56 [0.24, 2.56]	0.01 ± 0.16 [− 0.50, 0.21]
SyntEyes 120 months (n = 30, #201-230)	47.22 ± 3.95 [40.81, 58.74]	44.06 ± 3.63 [38.52, 51.14]	− 3.15 ± 1.38 [− 0.63, − 7.61]	0.82 ± 0.47 [0.22, 2.35]	− 0.03 ± 0.19 [− 0.66, 0.26]

#### Step 2 and 3: Optimize defocus and astigmatism target based on corneal aberrations

Custom routines (Matlab R2021b, The MathWorks, Inc) were developed to combine multiple sphero-cylindrical corrections with the natural corneal HOAs of each SyntEye. The sphero-cylindrical corrections were converted to lower order Zernike coefficients in the entrance pupil and added to the higher order terms (6th order, 4-mm pupil diameter). The optical quality was described in terms of the Visual Strehl of the Optical Transfer Function (VSOTF) as this metric has been shown to correlate best with logMAR visual acuity^25^. VSOTF was computed through-focus from + 5D to − 5D in 0.25D steps in eyes with RMS HOAs lower than 0.5 μm, and from + 10D to − 10D in 0.25D steps in eyes with RMS HOAs equal or higher than 0.5 μm. For different astigmatism corrections and orientations these ranges were from − 0.5D to − 5D in 0.25D steps (RMS HOAs < 0.5 μm) or from − 0.5D to − 10D in 0.25D steps (RMS HOAs ≥ 0.5 μm) for the astigmatism power, and from 0° to 175° in 5° steps for the orientation. The targeted defocus and astigmatism producing the maximum VSOTF was considered as the optimal sphero-cylindrical correction for a specific cornea. In addition, we analyzed the VSOTF that fully compensates the defocus and astigmatic terms (“zero target”) as a reference. The visual benefit was defined as the Visual Stehl of the optimized method (VSOTF_optim_) divided by the zero-target Visual Strehl (VSOTF_zero_).

#### Step 4: Customized eye models: ray tracing optimization (RTO) method for IOL power calculation

Based on the optimized refractive target from the previous step, the wavefront aberrations and residual refraction were calculated in the 4-surface model (4-mm pupil diameter), containing the anterior and posterior corneal surfaces, the shape of the IOL (assuming perfect centration and no lens tilt), the estimated lens position (i.e., anterior chamber depth + 0.3 times the lens thickness) and the axial length of the SyntEye. The IOL considered was the Precizon Toric IOL (OPHTEC BV, Groningen, The Netherlands) with a refractive index of 1.46. The resulting sphero-cylindrical correction producing the highest VSOTF was considered as the refractive target for IOL selection. Finally, the IOL power, cylinder and axis that matched the optimal sphero-cylindrical correction within 5% of the refractive error was selected.

### Data analysis

The ray tracing optimization (RTO) method for IOL power calculations were compared with the IOL power and cylinder calculated using the Saunders–Retzlaff–Kraff theoretical (SRK/T) formula using the A-constant of 118.6 recommended by the manufacturer. The Zernike defocus and astigmatism coefficients of the eye models were converted into power vectors (spherical equivalent M and Jackson cross-cylinders J_0_ and J_45_) to obtain the sphero-cylindrical notation of the refractive state. Finally, the tolerance of both methods to potential variations in IOL plane position (± 0.5D of defocus) and IOL rotation (± 5°) was also determined. For all analyses, the statistical significance was defined at p < 0.05. The prediction error was analyzed statistically using paired 2-tailed t test.

### Ethics declarations

The author(s) have made the following disclosure(s): P.P.-M.: Co-founder at 2 Eyes Vision, SL, outside of the current study. J.A.: Consultant for Staar Surgical, Johnson & Johnson and Alcon Laboratories, outside of the current study. A.V.Q.: CTO and co-founder at Azalea Vision, outside of the current study. J.J.R.: None.

## Results

### Effect of optimization

**Figure 2:**
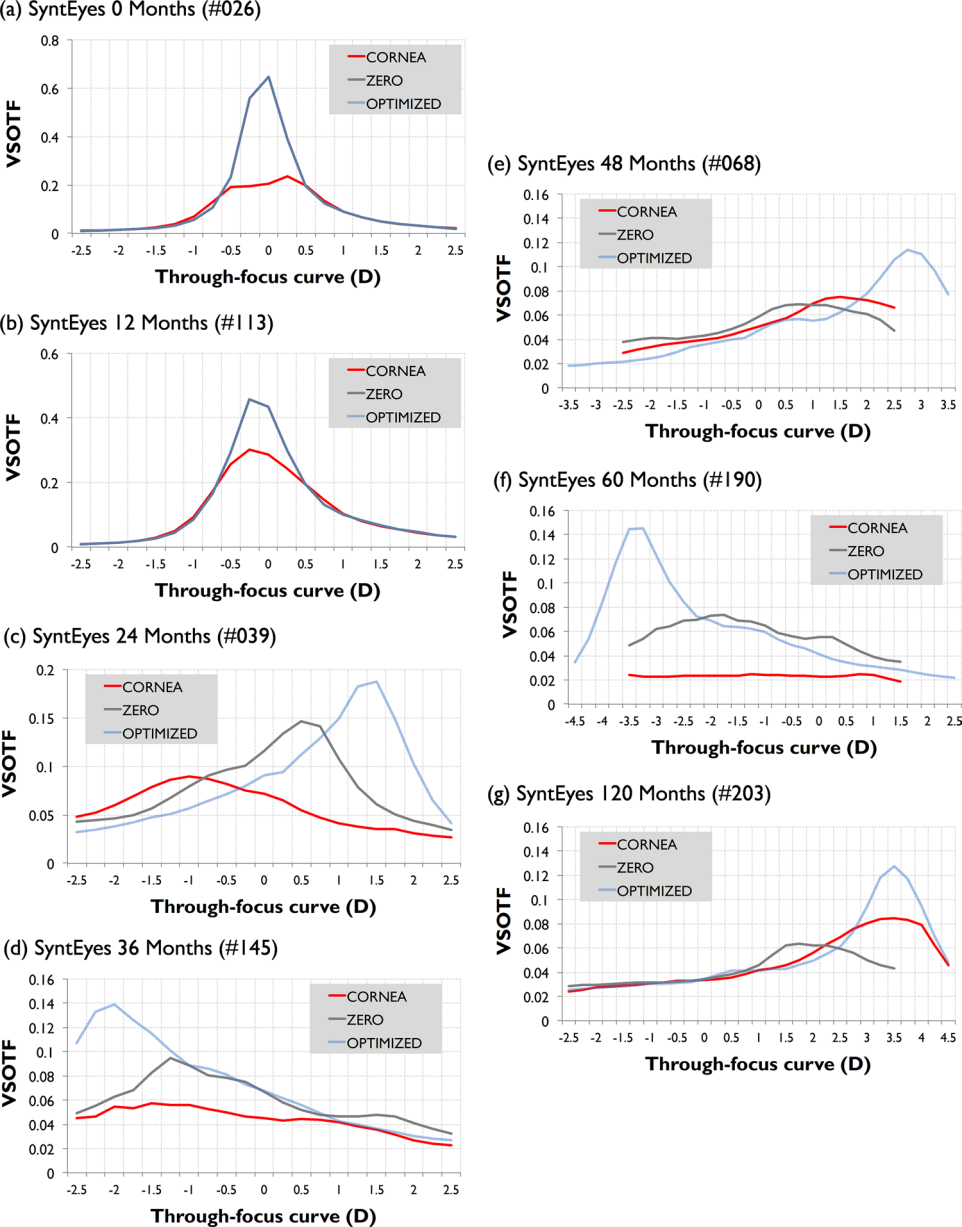
Through-focus Visual Strehl (VSOTF) for representative examples of each SyntEye group: uncorrected cornea (red), zero-target (grey) and optimized (light blue).

**Figure 3:**
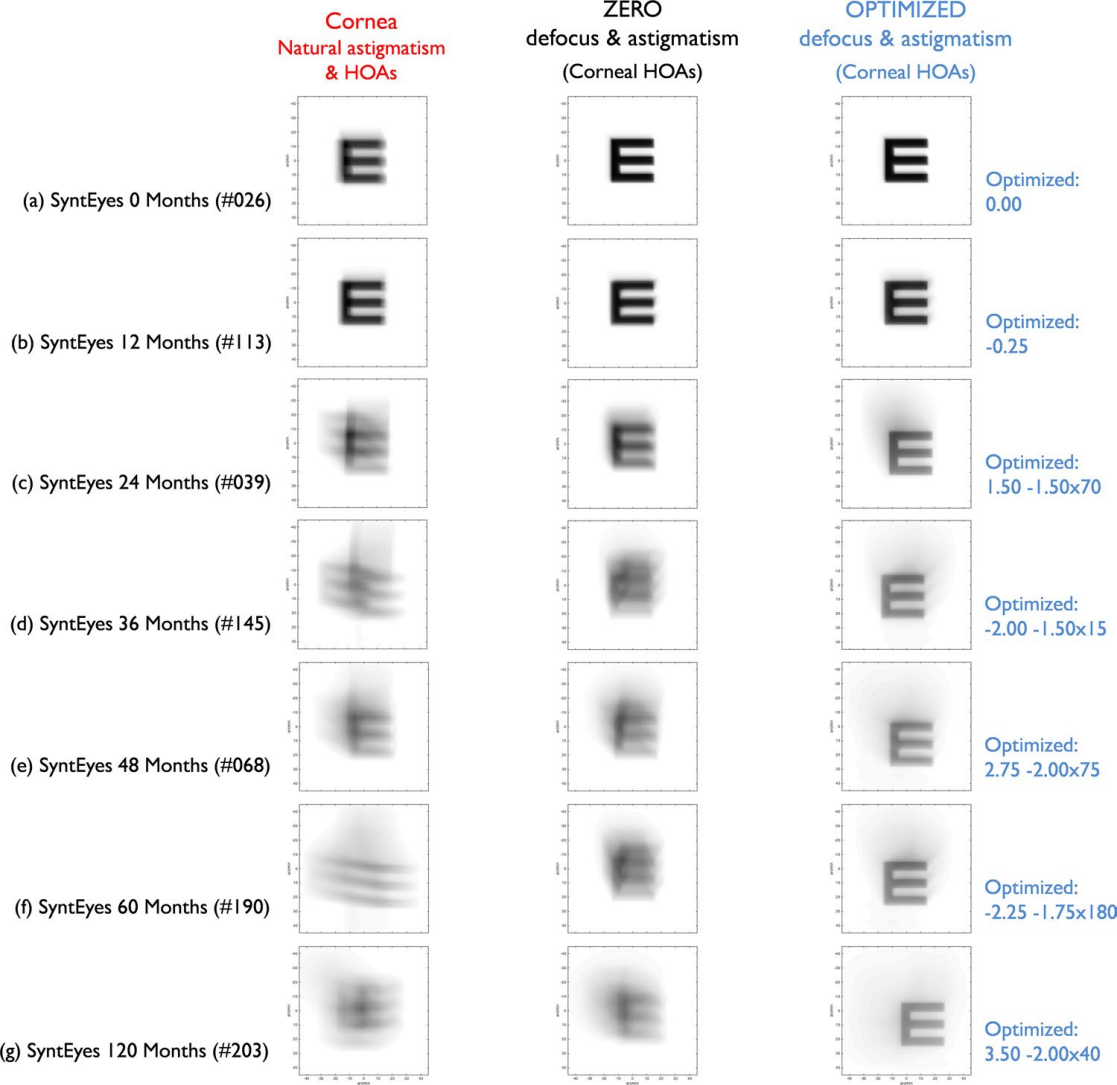
Theoretical simulations of 30 arc-min Snellen E-letters (4-mm pupil) for representative examples of each SyntEye group. Left column: convolved letter with the astigmatism and natural aberrations of the cornea. Middle column: convolved letter after full cancellation of defocus and astigmatism. Right column: convolved letter with the sphero-cylindrical correction that produced the best optical quality (peak Visual Strehl).

The through-focus optimization leads to a gradual improvement in VSOTF as the sphero-cylindrical correction approaches the optimal value (Fig. 2). It does so differently for the zero-target and the optimized sphero-cylindrical corrections, which has consequences for the simulated visual performance (Fig. 3). In mid- to late-stage keratoconus with an irregular cornea (i.e., from 24 to 120 months after simulated onset), the optimized defocus and astigmatism corrections outperformed the zero-target correction due to the amount of residual HOAs.

Figure 3 illustrates the simulated visual performance of the residual aberrations at the pupil plane for natural corneal astigmatism, zero-target correction and optimized-target using a convolved Snellen E letter (30 arc-min, 4-mm pupil) in seven representative examples: (a) SyntEyes 0 months (RMS HOAs: 0.11 μm), (b) SyntEyes 12 months (0.14 μm), (c) SyntEyes 24 months (0.41 μm), (d) SyntEyes 36 months (0.63 μm), (e) SyntEyes 48 months (0.72 μm), (f) SyntEyes 60 months (0.64 μm) and (g) SyntEyes 120 months (0.90 μm). These examples show that RTO generally produced the highest VSOTF and a higher visual quality.

**Figure 4:**
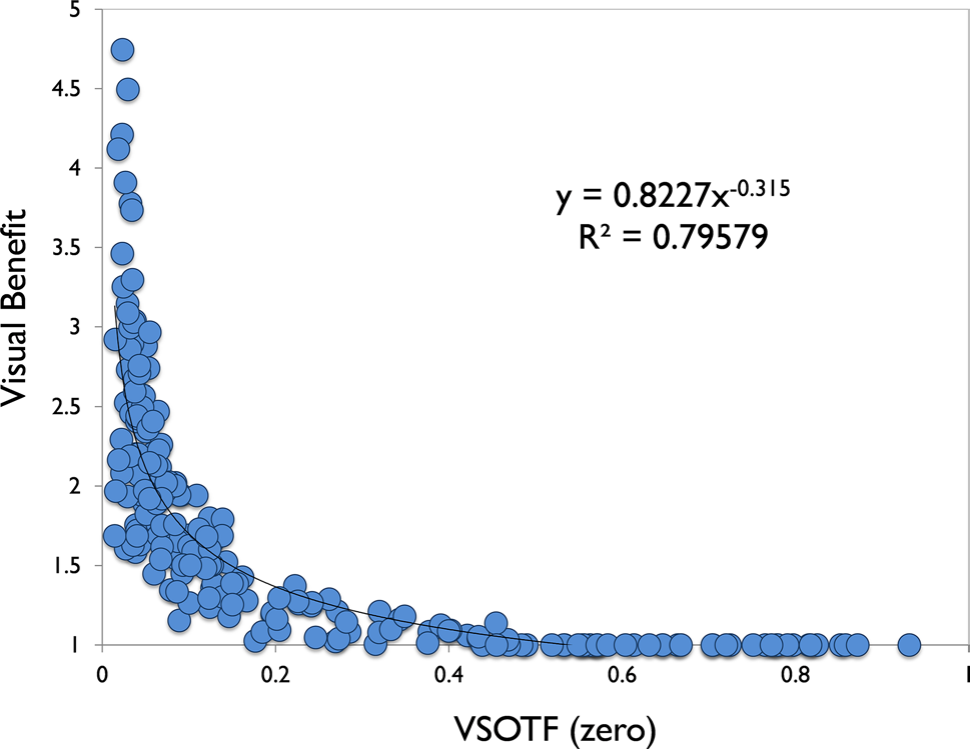
VSOTF (zero defocus and astigmatism) vs. Visual benefit, defined as VSOTF (optimized defocus and astigmatism) divided by VSOTF (zero defocus and astigmatism).

Looking at the visual benefit of the optimized correction in all SyntEyes, it is seen that corneas with more HOAs and a lower VSOTF_zero_ experienced a greater visual improvement with the RTO method (Fig. 4). This is also seen in Table 2, where it is shown that the percentage of eyes above the threshold for acceptable vision (i.e., VSOTF ≥ 0.12)^25^ is considerably higher for the RTO method.

**Table 2:** Optimized refractive target, visual benefit and comparison between the zero-target and optimized methods (average ± standard deviation [minimum, maximum]).

	Optimized refractive target			Visual benefit	Acceptable vision	
	M (D)	J_0_ (D)	J_45_ (D)		Zero—target (%)	Optimized—target (%)
SyntEyes 0 months	− 0.05 ± 0.12 [− 0.50, 0.00]	0.01 ± 0.04 [0.00, 0.22]	− 0.01 ± 0.02 [− 0.12, 0.00]	1.01 ± 0.04 [1.00, 1.21]	100	100
SyntEyes 12 months	− 0.18 ± 0.31 [− 1.50, 0.00]	0.03 ± 0.09 [0.00, 0.44]	− 0.02 ± 0.09 [− 0.36, 0.23]	1.06 ± 0.15 [1.00, 1.21]	100	100
SyntEyes 24 months	− 0.72 ± 2.18 [− 6.00, 2.50]	0.00 ± 0.68 [− 1.47, 1.47]	− 0.08 ± 0.71 [− 1.76, 0.92]	1.62 ± 0.62 [1.08, 4.49]	53.3	80.0
SyntEyes 36 months	− 0.21 ± 3.13 [− 10.25, 4.75]	− 0.22 ± 1.29 [− 3.43, 3.18]	0.40 ± 0.67 [− 0.85, 2.22]	2.06 ± 0.70 [1.00, 3.77]	20.0	63.3
SyntEyes 48 months	− 0.81 ± 3.85 [− 12.00, 4.50]	− 0.22 ± 1.47 [− 3.52, 3.52]	0.22 ± 0.99 [− 2.20, 2.20]	2.02 ± 0.62 [1.04, 3.91]	20.0	36.6
SyntEyes 60 months	− 1.12 ± 3.34 [− 8.50, 7.00]	− 0.06 ± 1.57 [− 3.52, 3.92]	0.10 ± 0.84 [− 1.98, 1.96]	2.07 ± 0.66 [1.14, 4.21]	16.6	53.3
SyntEyes 120 months	− 1.03 ± 3.64 [− 8.50, 7.75]	0.11 ± 1.44 [− 5.29, 1.71]	0.08 ± 1.15 [− 2.79, 2.94]	2.25 ± 0.92 [1.05, 4.75]	26.6	50.0

### IOL power calculation: estimation error

Once the optimized sphero-cylindrical corrections were available, they were introduced into the ray tracing optimization (RTO) IOL power calculation method. This leads to IOL powers ranging between 11.0 and 26.5D for the normal SyntEyes (0–12 months simulated progression) and 1.0–27.5D for the keratoconic SyntEyes (≥ 24 months simulated progression). The range on the cylinder corrections were 0.0–2.5D and 0.0–10.0D, respectively.

**Figure 5:**
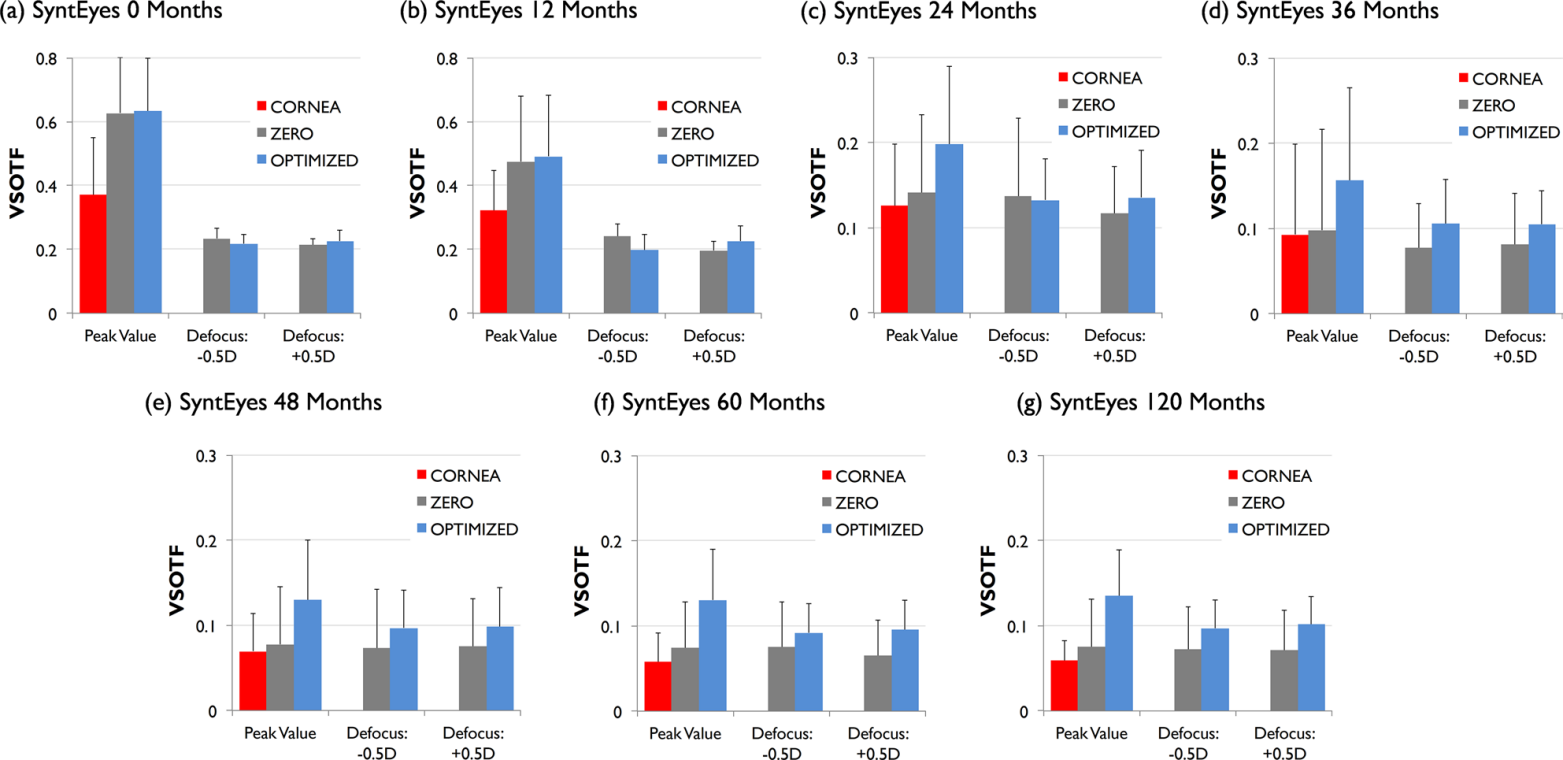
VSOTF magnitude for the original data (corneal astigmatism and HOAs, in red), zero target condition (zero defocus and astigmatism, in grey) and optimized calculation (sphero-cylindrical correction combined with the natural amount of HOAs, in light blue) and tolerance to ± 0.5D of defocus (zero target condition and optimized calculation).

Considered over all SyntEyes, the RTO method significantly increased the peak VSOTF from 0.15 ± 0.15 (original data: corneal astigmatism and HOAs) and 0.22 ± 0.24 (zero-target method) to 0.27 ± 0.22 (p < 0.001). The same was seen in each individual SyntEyes group (p < 0.001). Note that as the degree of keratoconus increased the differences between the original corneal data and the zero-target gradually became non-significant (Fig. 5, bottom row), highlighting the importance of taking the HOAs into account when estimating the IOL power and cylinder.

To test the stability of the optimized and zero-target corrections, an artificial defocus of ± 0.5D was induced. In both cases this led the VSOTF to decrease compared to the in-focus case (Fig. 5). For SyntEyes with few HOAs the loss in VSOTF was similar for both correction methods, but for the higher amounts of HOAs in moderate and advanced keratoconus the VSOTF of the optimized correction reduced significantly less than that of the zero-target correction (p < 0.001).

**Figure 6:**
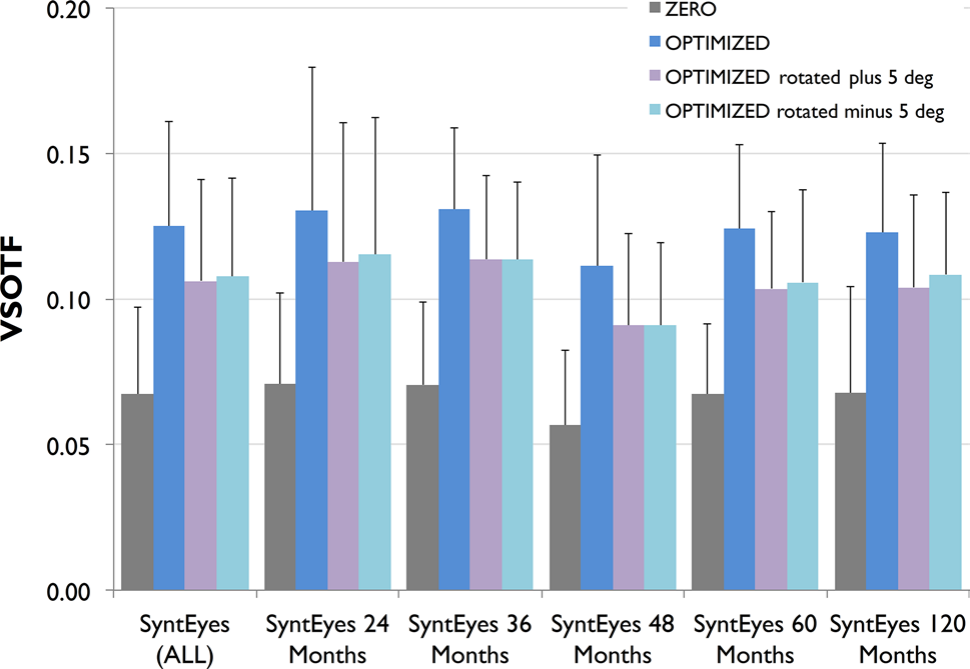
Tolerance to toric IOL rotation: VSOTF magnitude for the optimized calculation and zero target condition (zero defocus and astigmatism).

Figure 6 shows the influence of IOL rotation in Visual Strehl in those eyes with a different cylinder target (zero-target *vs.* optimized target) in the calculation, which would have a different tolerance to rotation. The analysis includes 94 of 210 eyes: 15 SyntEyes at 24 months, 22 SyntEyes at 36 months, 18 SyntEyes at 48 months, 18 SyntEyes at 60 months and 20 SyntEyes at 120 months. The impact of a potential toric IOL rotation (± 5 degrees) in visual degradation is much lower than the degradation induced by defocus (optimized *vs.* optimized rotated) and we found that on average the Visual Strehl after IOL rotation was higher (p < 0.001) than the Visual Strehl calculated with zero defocus and astigmatism as the final refractive target.

### Ray tracing optimization (RTO) vs. SRK/T

**Figure 7:**
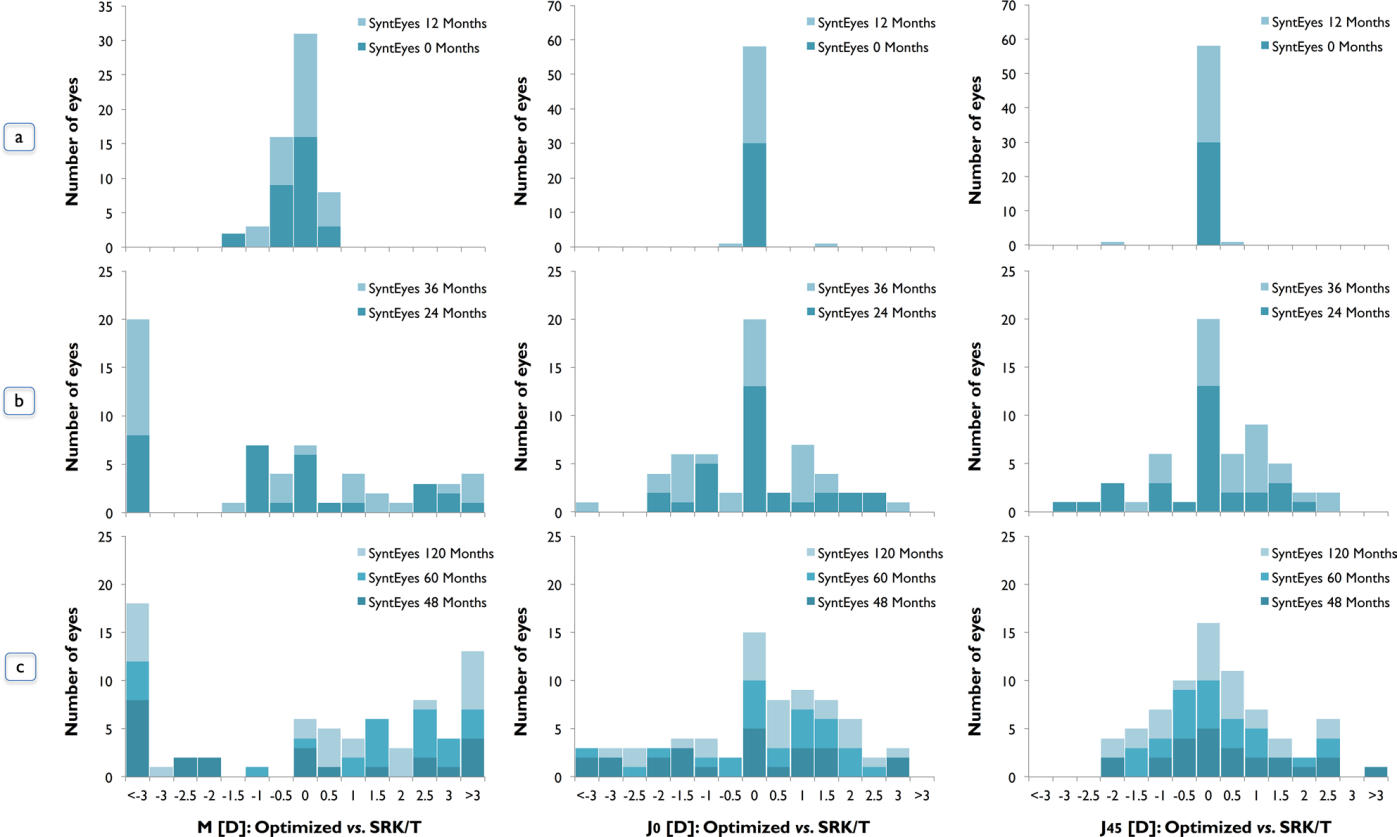
Difference in residual refractive error between RTO and SRK/T expressed as power vectors M, J_0_ and J_45_. (**a**) Regular astigmatism: Normal—SyntEyes 0 and 12 months; (**b**) irregular astigmatism and HOAs: SyntEyes with simulated progression of 24 and 36 months (mid-stage KC); (**c**) irregular astigmatism and higher amount of HOAs: SyntEyes with simulated progression of 48, 60 and 120 months (late-stage keratoconus).

The refractive target provided by the RTO and SRK/T shows a very good agreement in eyes with regular astigmatism and a low amount of HOAs (SyntEyes 0 and 12 months; Fig. 7a), where 91.66% of eyes agreed within ± 0.5D in IOL power calculation. For these groups, 26.66% of eyes will require a toric IOL implantation and only one eye would benefit from an optimized cylinder adjustment to obtain best visual performance.

Most SyntEyes with higher amounts of HOAs would need an optimization in power, cylinder and axis to achieve the best possible visual result (Fig. 7b,c) since only 21.05% of SyntEyes (simulated progression of 24 and 36 months, mid-stage KC) and 15.06% of SyntEyes (48, 60 and 120 months progression, late-stage KC) showed an agreement within ± 0.5D between RTO and SRK/T. For these cases, the power difference exceeded 3D in 42.10% (SyntEyes 24 and 36 months) and 42.46% (SyntEyes 48, 60 and 120 months), while the cylinder required adjustments larger than 3D in 12.28% and 24.65% of the cases, respectively. More than 80% of SyntEyes with 24–120 months of progression required a toric IOL to obtain best visual performance, especially those with larger amounts of corneal higher order aberrations. In 20/210 SyntEyes the IOL and/or cylinder power were not available in the Precizon toric catalogue, particularly eyes with an RMS HOAs higher than 1.1 μm (4-mm pupil) and corneal astigmatism higher than 5D. Those cases were excluded from this analysis.

## Discussion

Current methods of IOL power calculation generally fail in patients with irregular corneas, such as keratoconus or after corneal refractive surgery, because they rely on assumptions about the corneal shape or estimated lens position that may lead to postoperative refractive surprises^1,2,30–33^. One reason for this shortcoming is that IOL power calculations generally aim at minimizing the residual refractive error. The analyses above suggest that this may not always be ideal, however, since the position of best focus is influenced by corneal astigmatism and higher order aberrations, especially in keratoconus or after corneal refractive surgery. It may therefore be expected that such eyes could benefit from an optimized refractive target based on the three-dimensional shape of the anterior and posterior corneal surfaces, rather than simplified parameters such as keratometry^34–36^.

This work introduced such a customized IOL selection based on virtual ray tracing, keeping in mind that certain amounts of lower and higher order aberrations may interact favourably to improve visual performance by enhancing the IOL selection and prediction of the refractive outcome^25–27^. Our results suggest that the ray tracing optimization (RTO) method provides the greatest benefits for corneas with larger amounts of higher order aberrations, with 0.35 μm for a 4-mm pupil diameter (corresponding to 0.95 μm for a 6-mm pupil) as the RMS HOAs threshold for a manifest improvement in the IOL power calculation.

Our analysis expands those of previous ray tracing modules for IOL power calculation, such as e.g., Olsen’s PhacoOptics (IOL Innovations ApS, Aarhus, Denmark) or Okulix (Okulix, Dortmund, Germany) that model the IOL to determine the effective focal length matching the axial length (i.e., zero-target). Given the generally asymmetric nature of corneal topography in keratoconus and in post-refractive surgery eyes, the use of higher-order Zernike coefficients seems better suited for an optimized analysis as their orthogonality permits calculating the refractive power vectors. Furthermore, Visual Strehl, an optical quality descriptor that combines the impact of the optical aberrations with a measure of neural performance, is a good optical predictor of visual acuity^25^.

Pseudophakic eyes have the distinct advantage that they are easier to model than phakic eyes, since the refractive index and curvatures of the IOL are known—at least to the manufacturer, who are rarely willing to share these details—but also present a challenge as the residual refraction depends in part on the postoperative IOL alignment and position^8^, which in turn is determined by the capsular healing process. To this end, we analyzed the impact of a potential shifts and rotations away from the anticipated IOL position and orientation, which demonstrated a higher tolerance to loss in VSOTF for the RTO method compared to zero-target.

Previous studies showed that using traditional formulas will bring only 26–35% of eyes to within 0.5D of the targeted astigmatism in regular corneas^37^, with a post-operative residual astigmatism of − 0.64 ± 0.43D^15^, − 0.71 ± 0.43D^16^ and − 1.03 ± 0.79D^38^. Other studies described that IOL power calculation is considerably less accurate in keratoconus than in typical eyes^17,18,30^. The SRK/T formula showed the highest accuracy for IOL power calculation in patients with moderate keratoconus, but only 36–44% of eyes achieved a result within 0.5D of the final predicted refraction^17,39^. To improve the refraction prediction in eyes with keratoconus, two dedicated formulas were developed, Kane keratoconus^30^ and Holladay 2 with keratoconus adjustment^31^. Although the Kane formula provided more accurate predictions compared with the Holladay method (50%-Kane vs. 27.4%-Holladay of eyes within 0.5 D), the predictability of the formula is lower than in normal eyes and requires further refinement, particularly in moderate and advanced stages with high levels of corneal aberrations. This underlines once more the need for new IOL calculation strategies.

One of the limitations of our study is that the computational processing time per eye was long since it requires the evaluation of multiple possible sphero-cylindrical corrections to determine the final IOL power and cylinder (e.g., 40 min per eye for eyes with RMS HOAs > 0.5 μm, 4-mm pupil). Another aspect is that the analysis used SyntEyes instead real eyes. SyntEyes for normal eyes and developing keratoconus were previously validated with real eyes^28^ and offer a complete database of anterior and posterior corneal elevation points with increasing high-order corneal irregularities. This model for keratoconus progression allows the development of realistic personalized eye models to simulate how an increasing magnitude higher order corneal aberrations affects the final IOL power and cylinder selection. The analysed HOAs were also encountered clinically^29^ and the methodology could be directly translated into a clinical application by using the anterior and posterior raw elevation data of commercial corneal tomography devices.

Multiple studies described the excellent repeatability and reproducibility of corneal topography measurements provided by different types of imaging systems^40–43^. There is some kind of controversy, however, about the reported repeatability for some corneal parameters after corneal refractive surgery^44^, as well as in patients with dry eye^45^, keratoconus^46^ and patients at the typical age for cataract^47^. The statistical uncertainty of the elevation measurements also increases toward the peripheral area, but the central 6-mm region may be considered as reliable^48–51^. This was confirmed by Guirao and Artal^52^, who described an RMS error of 0.05 μm (4-mm pupil) and 0.2 μm (6-mm pupil) for the estimation of the corneal wave aberration from corneal elevation data. SyntEyes assume a corneal diameter of 6.5 mm, which only marginally larger than this region.

Hence, the choice of the 4-mm central region will potentially allow (i) acquiring more reliable corneal elevation data (i.e., fewer missing data points due to eyelids, etc.), (ii) increasing the accuracy of the estimated corneal wavefront aberrations^52^, (iii) reducing measurements errors due to blinking, eye movements, poor centration or alignment^49^, and (iv) targeting possible measurements of keratoconus patients with intracorneal ring segments, avoiding the measurement distortions introduced by the segment itself^29^. Besides, the typical pupil diameter is around 4 mm in 50 and 79 year-olds for a luminance level of approximately 220 cd/m^2^^53^, the surface design of most IOL models are optimized for a pupil size of 4–5 mm^54,55^ and retinal image blur caused by diffraction is negligible when compared to the effects of aberrations for pupil diameters greater than approximately 3–4 mm^56^.

In conclusion, the proposed ray tracing optimization method incorporates the interactions between corneal aberrations and the IOL design, allowing for realistic simulations of defocus, astigmatism, and higher-order aberrations, to accurately calculate the required IOL power and cylinder. Such patient-specific IOL selection based on virtual ray tracing has great potential, but still needs prospective clinical validation with real cataract patients.

## Data Availability

The datasets used and analysed during the current study are available on reasonable request from Pablo Pérez-Merino (pablo.perezmerino@ugent.be).
